# Keratoacanthoma versus Squamous-Cell Carcinoma: Histopathological Features and Molecular Markers

**DOI:** 10.3390/dermatopathology11040029

**Published:** 2024-10-08

**Authors:** Hisham F. Bahmad, Kalin Stoyanov, Teresita Mendez, Sally Trinh, Kristy Terp, Linda Qian, John Alexis

**Affiliations:** 1 Arkadi M. Rywlin M.D. Department of Pathology and Laboratory Medicine, Mount Sinai Medical Center, Miami Beach, FL 33140, USA; john.alexis@msmc.com; 2 Herbert Wertheim College of Medicine, Florida International University, Miami, FL 33199, USA; 3 Department of Pathology, Herbert Wertheim College of Medicine, Florida International University, Miami, FL 33199, USA

**Keywords:** keratoacanthoma, squamous-cell carcinoma, molecular pathways, pathology, histology

## Abstract

Considerable controversy exists within the field of dermatopathology in differentiating keratoacanthoma (KA) from squamous-cell carcinoma (SCC). KAs are rapidly growing, benign squamous tumors that are typically well differentiated. This controversy stems from the diverging perspectives on the management, classification, and diagnosis of each entity. Many believe that KAs are benign neoplasms in which intervention may be unnecessary since they are self-limiting and resolve on their own. On the other hand, SCC needs to be treated, as it carries significant morbidity and mortality risks. Early diagnosis and treatment are vital to prevent serious consequences of SCC. Nevertheless, KAs may resemble SCC grossly and microscopically. Various ancillary tests, including immunohistochemical (IHC) staining, have been proposed to differentiate between these entities, though mixed patterns of expression can limit the diagnostic utility of these techniques. Research into this topic is ongoing, with newer genetic and molecular findings illuminating the previously difficult-to-understand aspects of KA and increasing our understanding of this entity. In this review, KA and SCC will be compared along the lines of histological features, genetic, immune, and molecular markers, differential diagnosis, and management to clarify the similarities, differences, and misconceptions about both entities.

## 1. Introduction: Definition and Epidemiology

Keratoacanthoma (KA) is a common, potentially under-reported skin tumor that has intrigued dermatologists and dermatopathologists alike for decades. Different terminologies have been used in the past to describe this entity, including “molluscum sebaceum”, “pseudotumor”, “regressing tumor”, and “self-healing squamous-cell carcinoma” (SCC), all of which reflect the controversy revolving this lesion [1]. KA is a benign, rapidly growing cutaneous neoplasm that often presents as a solitary, dome-shaped papule or nodule on sun-exposed skin, most commonly on the face, neck, and extremities. It was first described by Sir Jonathan Hutchinson in 1888, who classified it as a form of epithelial cancer [2]. Clinically and histologically, it shares similarities with SCC, making accurate diagnosis crucial to guide appropriate management. The unique clinical course of KA, characterized by rapid growth over a few weeks to months followed by spontaneous regression, sets it apart from other skin lesions and underscores the need for precise histopathologic assessment.

The incidence of KA may be underestimated due to misdiagnosis as SCC, under-reporting, or spontaneous regression. A 2014 study conducted in the United Kingdom and Ireland found a wide SCC/KA ratio range among pathologists due to diagnostic variations [3]. KA is more common in fair-skinned individuals and is more prevalent in men than women. Sun-damaged skin increases the risk, with the peak incidence shifting from ages 50–69 years in the 1990s to 65–71 years today [1,4,5].

### 1.1. Basic Histopathologic Features of Keratoacanthoma

Regarding its histopathologic features, KA typically appears symmetric. It has an exo–endophytic architectural configuration, presenting as a crateriform nodule with well-defined borders and invaginated infundibular-like components [6,7]. It contains a central keratin plug (keratin-filled crater) with “overhanging epithelial lips” and a normal overlying epidermis that surrounds the lesion [6,7]. The transition between the lesion itself and the surrounding epidermis is abrupt, not gradual [8]. KA is composed of large squamous cells with hyperchromatic to vesicular nuclei and prominent nucleoli, with abundant, eosinophilic cytoplasm [7]. When a KA-like tumor shows asymmetry, irregularities in the overhanging epithelial lips, or variability in the organization of the aforementioned squamous cells, the diagnosis becomes less clear and may lead to non-committal terminology such as “SCC-like” or a “KA with SCC components” [6], with consequent uncertainty in determining the clinical course and appropriate therapy. KAs may also demonstrate perineural invasion and intravascular spread, which do not deem them malignant per se [9,10]; nevertheless, they might have increased risk for local recurrence. In fact, KAs that have perineural invasion or intravascular spread also regress just like any other KA, meaning that these are usually incidental findings [10].

### 1.2. Histologic Phases of Keratoacanthoma

Since KAs regress, their growth may be subdivided into three phases, each with identifiable histological features. These phases are (1) proliferation phase, (2) maturation phase, and (3) regression or involution phase.

#### 1.2.1. Proliferation Phase

**Figure 1:**
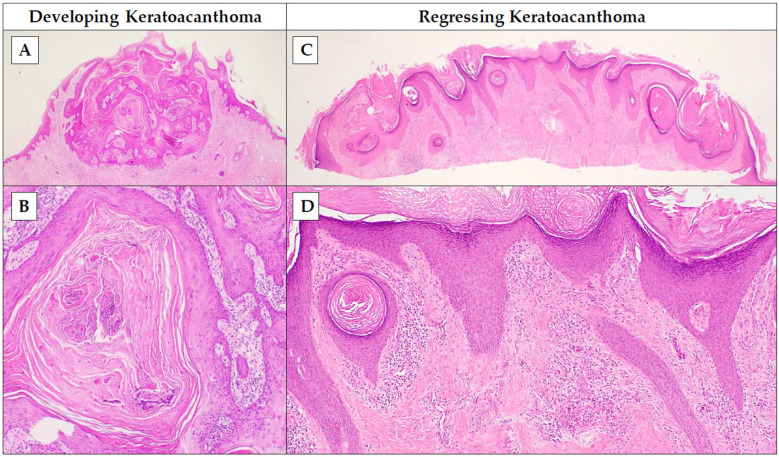
Histopathologic features of keratoacanthoma. *Developing keratoacanthoma* includes two phases: the proliferation phase (where the tumor is small, primarily solid, with distinct infundibulocystic structures that have not yet coalesced into a central keratin plug, containing islands of laminated keratin with a ground-glass appearance) and the maturation phase (which demonstrates an exo–endophytic squamous proliferation with a central keratin plug, overhanging epithelial lips, and compact keratinization). *Regressing keratoacanthoma* shows a “hollowing out” lesion with loss of central keratin plug, perilesional lymphohistiocytic infiltrate, and increasing fibrosis (H&E; magnification: (**A**,**C**): 40×, (**B**,**D**): 100×).

The early proliferation phase of KAs is characterized by a small, primarily solid tumor that has several distinct infundibulocystic structures that have not yet coalesced into a central keratin plug [7,8]. These structures contain islands of laminated keratin with a ground-glass appearance, which may merge with one another [7] (Figure 1). Notably, there should be no nuclear atypia within the center of these structures, though some atypia in the periphery of the tumor may be allowed [7]. Nuclear atypia in KAs tends to become less prominent as the lesion matures [11]. Inflammation may be seen with a mixture of histiocytes, eosinophils, neutrophils, and plasma cells being present [8]. Interestingly, the early proliferative phase does not determine the level of growth that the lesion will ultimately exhibit nor the size it will reach at maturity [1]. Thus, a KA which appears small in the early proliferative phase may grow up to 20 cm (as in cases of the KA centrifugum variant, also known as KA centrifugum et marginatum [12]) without any obvious histological predictors of its final size [1].

#### 1.2.2. Maturation Phase

The maturation phase has more defined features typical of KAs, such as the characteristic exo–endophytic architecture with a central keratin plug, the overhanging epithelial lips, and compact keratinization [7] (Figure 1). In this phase, there may be significant variability of cellular appearance and atypia within the lesion, with a spectrum. The spectrum describes the cytologic distribution of nuclear atypia, with most of the pleomorphic cells being located in the most distant fringes [8]. The majority of KAs are captured during this phase, as this is typically the time when the lesion is clinically concerning [8].

#### 1.2.3. Regression Phase

The regression phase presents with a “hollowing out” of the lesion, with the tumor losing its characteristic central keratin plug [7,8]. The lesion begins to resemble an empty bowl as the keratin plug clears and the epithelium thins [7]. Perilesional dermal inflammation, particularly lymphocytic infiltrate, and cicatricial fibrosis complete the histological picture [7,8] (Figure 1).

### 1.3. Regression of Keratoacanthoma

The spontaneous resolution of KAs is believed to be related to their origin from hair follicles. Hair follicles naturally cycle through anagen, catagen, and telogen phases, leading to cyclical growth, regression, resting and hair shedding. KAs have been shown to express high levels of apoptotic genes and pathways that drive the characteristic regression of KAs [13]. In particular, it has been shown that KAs have upregulated levels of classically pro-apoptotic genes, such as *BAK* and *BAX*, while having downregulated levels of anti-apoptotic genes, such as *BCL2* and *BCL2*-*like 1* (encoding Bcl-xL protein) [13]. Several proposed and tested proteins, genes, and pathways regarding the regression of KAs will be discussed in further detail in the following section. 

While KAs spontaneously regress and involute by definition, SCC does not spontaneously involute [14]. Thus, SCC and various other entities must be ruled out, so that a patient is not falsely reassured that they have a benign tumor and end up with untreated SCC.

### 1.4. Molecular Pathways Contributing to the Formation of Keratoacanthoma

In considering the growth patterns of KAs, several alternative pathways become activated and/or repressed in the different phases. For example, the Wnt/β-catenin pathway is activated during the growth phase and inactivated during the regressing phase [1,15]. It is essential to consider the reoccurring theme of this tumor’s hair follicle-derived nature, as Wnt signaling is upregulated during the onset of normal hair growth [16]. Thus, it is not surprising to see this also occurring during KA growth. Targeting this pathway has been proposed and tested as a therapeutic approach to facilitate a speedier regression of KAs as discovering a way to inhibit Wnt signaling will promote regression [16]. This theory has been proven in mouse studies and may well become an alternative treatment for KAs in the future [16].

As discussed, since the major hypothesis behind the involution of KAs revolves around their similarities to the hair follicle, we expect that similar genes and their corresponding proteins that are upregulated in aging hair would be present in KAs. Several upregulated proteins such as matrix metalloprotease-1 (MMP1), S100 calcium-binding protein A8 (S100A8), and tumor protein 63 (TP63) have been associated with the mass apoptosis that occurs in the final phases of KAs [13]. MMP1 activity, for example, is associated with collagen fragmentation, which is recognizable during the regressing phase of KAs [17]. Another example is cyclin-dependent kinase inhibitor p27, which is uniquely present exclusively during the regressing phase of KAs and absent during growth [18].

### 1.5. Pitfalls in the Diagnosis of Keratoacanthoma

A simple error that may impact the diagnosis of KA versus SCC is an inadequate biopsy. It is essential to take an adequate sample by excising the KA in its entirety. As previously discussed, the distinct components and architecture of KA provide superior diagnostic utility compared to mere cytology; thus, it is vital not to miss these architectural elements due to an inadequate biopsy [1,8].

The cooperation between dermatopathologists and dermatologists is crucial in preventing such diagnostic pitfalls. Dermatologists play a key role in obtaining an appropriate biopsy sample, and direct communication with dermatopathologists can ensure the sample is optimal for histopathological evaluation. Close collaboration enables a more informed clinical context and correlation, which can reduce the misinterpretation of histologic findings. Studies have reported that dermatopathologists may over-report SCC when receiving an inadequate sample, further complicating the differentiation between KAs and SCCs [3].

Another cardinal diagnostic pitfall is failing to understand that a KA may look morphologically like a SCC and vice versa. This may lead to an over-reliance on immunohistochemical (IHC) staining, which remains problematic as no definitive stain exists to differentiate these lesions. Thus, a multifaceted approach should be utilized, in which the tissue sample is carefully re-examined, and multiple stains may have to be utilized to help narrow the diagnosis.

## 2. Differential Diagnosis of Keratoacanthoma

Several crateriform nodular or papular lesions may appear similar to a KA. These include lesions that appear similar to SCC, as well as inflammatory or infectious diseases [1]. Infundibular SCC, Bowen disease, verrucous carcinoma, seborrheic keratosis, actinic keratosis, KA-like SCC, and the controversial “KA with malignant transformation” may all fall in the differential diagnosis to consider when assessing the patient’s tissue [1,19] (Table 1). KAs may even resemble large-cell lymphomas and amelanotic melanomas [1]. Regarding infectious diseases, KAs may resemble sporotrichosis, cryptococcosis, blastomycosis, and molluscum contagiosum [1]. Regarding inflammatory diseases, KAs may resemble hypertrophic forms of discoid lupus erythematosus and lichen planus, halogenoderma, and prurigo nodularis [1].

To begin the discussion of the two entities, one must first mention an important precursor of SCC, actinic keratosis (AK). AK is associated with prolonged UV light exposure resulting in significant sun damage and progression to SCC in a small subset of lesions. Typically, AK presents as a scaly, whitish, sandpaper-like lesion that can evolve into erythematous brown papules. Histologically, actinic keratosis is characterized by an altered cornified layer and a stratum malpighii that ranges from atrophic with a loss of rete pegs to hyperplastic, with elongated rete pegs. Parakeratosis is typical, with nuclei retained within the stratum corneum, although an orthokeratotic variant also exists.

On higher magnification, one typically observes atypical keratinocytes in the basal epidermis, which appear jumbled and disorganized, often referred to as “windblown”. Importantly, atypia in AK does not extend through the full thickness of the epidermis. Additionally, the dermis of AK is characterized by solar elastosis, a result of chronic sun exposure.

One distinctive feature of AK is its tendency to spare adnexal openings, such as sweat ducts and hair follicles. This sparing results in the presence of normal-appearing keratinocytes surrounding these openings, along with a normal corneal layer above them, effectively halting parakeratosis and atypia at these sites.

SCC is the second most common type of non-melanoma skin cancer and may have an aggressive clinical course. The epidermal component may exhibit full-thickness atypia of keratinocytes (“SCC in-situ”). A less common but distinctive pathological feature of SCC is cyst-like keratotic structures known as keratin pearls. Local invasion into the dermis may be associated with necrosis and overt keratinization, depending on the degree of differentiation of the tumor. Clear cell changes may be observed due to glycogenation of the keratinocytes, and mitotic figures may also be apparent.

This is in contrast to KA, which typically displays three phases of progression, the first of which is proliferative and fast-growing, the second of which is well developed, stationary, and also referred to as the mature phase, and the third and final of which is characterized by spontaneous regression. Because of these three predictable phases, KA is regarded as a benign entity. The second phase shares the most histologic characteristics with SCC and is the phase most frequently biopsied.

A mature KA lesion highlights exo–endophytic architecture, a keratotic plug, and overhanging epithelial lips [7]. The endophytic component features large pale pink cells with nuclear atypia or mitotic figures predominantly observed in the peripheral areas. Some of these histologic findings pose the primary challenges in distinguishing between KA and SCC. Due to this, the identification of key histologic features becomes crucial for differentiating KA from SCC.

One such key histologic feature, as stated by Mandrell et al., is the presence of nuclear pleomorphisms and mitotic figures, which are typically concentrated in the periphery of the deep component in KA [8]. In contrast, SCC tends to exhibit mitotic figures scattered throughout. Mandrell et al. further outlines other important histological features that are crucial for distinguishing between these two tumors [8]. For instance, factors such as symmetry, the presence of a keratin plug, an abrupt transition zone between the tumor and the epidermis, a deep fibrotic band beneath the lesion, and intraepithelial neutrophilic microabscesses all support a diagnosis of KA. Conversely, asymmetry, a gradual transition zone, and fibrosis surrounding the tumor nests are more frequently observed in SCC. Additionally, the contiguous presence of AK is often seen in SCC, whereas in cases of KA, if AK is present, it is not typically contiguous.

Studies have shown that molecular distinctions have begun to emerge between some forms of KA and SCC [20]. In one study, SCC demonstrated a greater abundance of anti-apoptotic proteins. In other studies, one form of KA, known as Ferguson-Smith syndrome (multiple KAs, i.e., multiple self-healing squamous epitheliomas (MSSEs))—a rare, inherited skin disease that causes the development of many tumors that resemble squamous-cell carcinoma and which typically occurs in young adolescents—has been associated with a high incidence of *TGFBR1* mutations, whereas SCC has shown fewer documented instances of such mutations [20,21]. SCC has also been subject to molecular investigations through exome and targeted sequencing, revealing that 82% of its mutations are in *NOTCH1*/*2* genes. Additionally, array comparative genomic hybridization has been employed to detect chromosomal copy number changes, showing that KA exhibits aberrations in different chromosomes compared to SCC. Therefore, KAs can exist in the context of genetic syndromes (such as Ferguson-Smith syndrome) or triggered by various mechanisms such as Koebner from trauma or laser in the form of eruptive squamous atypia.

Li et al. demonstrated recurrent aberrations in KAs on chromosomes 17, 19, 20, and X in about a third of cases [22], whereas recurring aberrations in SCC were found on chromosomes 7, 8, 10, 13, 17, and X, with losses on certain regions of 17p and 17q [13]. Furthermore, multiple studies have noted that new therapies targeting *BRAFV600* and the hedgehog pathway have been associated with the development of SCC and KA-like lesions [23]. For example, studies on sorafenib (kinase inhibitor drug approved for the treatment of advanced renal cell carcinoma, advanced hepatocellular carcinoma, *FLT3-ITD* positive acute myeloid leukemia (AML), and radioactive iodine-resistant advanced thyroid carcinoma) reported two isolated cases of SCCs of the skin associated with taking this drug [24]. In another study, 9 out of 131 patients (6.8%) receiving sorafenib developed SCCs and/or KAs [25]. In addition, cases of SCC and KA-like lesions have been reported post-treatment in patients taking vemurafenib (BRAF inhibitor initially designed to treat malignant melanomas) [26]. Further investigations into these SCC and KA-like lesions have revealed a high prevalence of *RAS* mutations before the initiation of *BRAF* inhibitors. It is hypothesized that the activation of the mitogen-activated protein (MAP) kinase pathway, triggered by *BRAF* inhibitors, leads to cell proliferation and the emergence of these tumors several weeks after therapy is started [26]. In fact, it is recommended now to use vemurafenib with caution in patients with prior or concurrent cancer, particularly those associated with *RAS* mutations. Screening for other malignancies should begin prior to treatment with vemurafenib and be repeated during treatment as indicated [27].

## 3. Distinguishing Keratoacanthoma from Squamous-Cell Carcinoma

**Figure 2:**
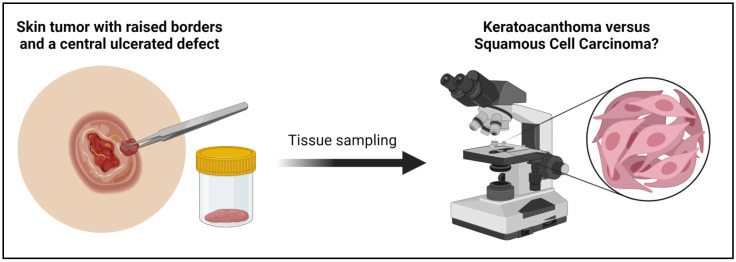
Schematic drawing of a skin tumor being sampled to make a histopathologic diagnosis of keratoacanthoma versus squamous-cell carcinoma.

Distinguishing between KA and SCC can be challenging as they may sometimes exhibit overlapping histological features (Figure 2). KAs typically exhibit symmetry and epithelial lipping, whereas SCCs may show ulceration, mitoses, and a random distribution of pleomorphic cells. However, the histological distinction between the two entities is not always clear.

### 3.1. Histologic Features and Key Criteria of Keratoacanthoma and Squamous-Cell Carcinoma

KAs are relatively common and arise from hair follicles. They may exhibit rapid enlargement and spontaneous regression [7]. Key criteria of KAs include an exo–endophytic architecture, symmetric and well-defined outline, overhanging epithelial lips with normal epidermis, and a central keratinous plug with a triphasic pattern of evolution including proliferative, stabilized, and regressive phases which reflect those of the hair cycle [7,28]. Those phases may show enlarged, pink, eosinophilic cells with ground-glass cytoplasm lacking nuclear atypia, epidermal lips partially covering the top of the KA, keratin-filled crater with well-differentiated squamous cells, and possible lichenoid inflammatory reactions depending on the stage [6,20,28,29]. SCC, on the other hand, is malignant with metastatic potential and has notable features of irregular nests or sheets of neoplastic keratinocytes that show dermal invasion along with increased thickness of the stratum corneum, parakeratotic cells, atypical hyperplasia of the basal epidermal layer, and possible neovascularization [30].

Several histological features are of particular importance in distinguishing between KAs and SCCs:

- KAs exhibit a significant degree of symmetry and epithelial lipping [1]; SCCs do not.
- KAs do not tend to spread beyond the level of the sweat glands [31], whereas SCCs may spread further due to their invasive nature [31].
- SCCs often exhibit more cytologic atypia than KAs.
- Ulceration and mitoses favor SCC over KA [1].
- Atypia in KA is typically located in the periphery [8]. In contrast, SCC has a more haphazard distribution of pleomorphic cells [8].

A diagnosis of SCC will have several key criteria that separate it from KAs. Several findings should strongly influence suspicion of SCC. A finding of ulceration or crusting on the sample increases the chance of SCC by a multiplicative factor of 24.7 [31]. There are certain pathognomonic findings, such as keratin pearls in SCC, which KAs lack [31]. SCCs tend to have a higher degree of pleomorphism, specifically including nuclear hyperchromasia, mitotic figures, and variability in cell size [31]. A rule of thumb is that the more atypical the features are, the higher the likelihood of the lesion being a SCC rather than a KA—with the caveat being that this is a rule of thumb. 

### 3.2. Immunohistologic and Immunocytologic Markers Distinguishing Keratoacanthoma and Squamous-Cell Carcinoma

It is important to note that no single IHC stain can definitively distinguish between KA and SCC, with a great variety of potential markers being evaluated [1]. Namely, the presence of several markers such as BDCA2, IL-27, TUNEL, Ley, Cyclin A, Cyclin B, P2X7, p50, E-cadherin, Cortactin, Lectin, and Syndecan-1 are favorable in identifying KAs compared to SCC [1]. High levels of several other markers, such as CD1a and Hsp60, have been found to favor SCC compared to KA [32]. This list is not exhaustive, but its length is testimony to the level of investigation performed (Table 2).

#### 3.2.1. CK17 and Ki67

To provide an example of an approach that may be used in clinical diagnosis, we will use a scenario in which IHC stains can be used to distinguish KA versus SCC in more straightforward ways. The first example will employ CK17 and Ki-67, which have recently been found to be effective in separating typical presentations of SCC and KA [33]. Ki-67 is a cell cycle-regulating marker protein that has been seen in squamoproliferative lesions [33]. CK17 is a keratin protein expressed in the outer root sheath of hair follicles and has been associated with disease progression of SCCs [33]. In one study, Ki-67 was discovered to have a diffuse staining pattern in SCCs (81% sensitivity and 100% specificity) while CK17 was found to have a central staining pattern in KAs (92% sensitivity and 70% specificity) [33]. Yet lesions with features of both SCC and KAs had mixed patterns [33] and the difficulty lies with tumors that are neither central nor diffuse in their staining or are a mix of the two [33]. Other studies found that, generally, there was more expression of Ki-67 in SCCs than KAs [37,38,39,40,41,42]. 

#### 3.2.2. Anti-P2X7

Another IHC stain, anti-P2X7, has also been used successfully in differentiating between KA and SCC, particularly in atypical presentations [43]. Anti-P2X7 in SCC shows extensive staining from the surface of the epidermis all the way to the margin where the tumor meets the dermis [43]. KAs do not have this staining distribution, and thus, the use of anti-P2X7 is helpful when morphology is not definitive [43]. This illustrates how a two-step approach can be used to distinguish KA and SCC.

#### 3.2.3. CD123

A study analyzed the number and distribution of plasmacytoid dendritic cells (PDCs) using CD123 in 66 KA and 63 SCC cases. Although KA had a slightly higher number of PDCs (14.2 ± 15.3) compared to SCC (11.2 ± 15.3), the difference was not statistically significant. Additionally, the mean number of PDC clusters, the number of intratumoral PDCs, and the relative proportion of PDCs within the inflammatory infiltrate showed no significant differences. These findings contrast with previous studies [44].

### 3.3. Genetics and Molecular Alterations in Keratoacanthoma versus Squamous-Cell Carcinoma

Recent research focusing on the molecular perspective of KA and SCC reveals that they are molecularly distinct [13]. In addition to providing another means to differentiate KA and SCC, genetic analysis highlights KAs’ pathways, answering long-debated questions about how similar KAs and SCCs are and whether KAs are hyperplastic or neoplastic [13]. 

KAs are neoplasms, according to molecular and genetic analysis, which have unique molecular mechanisms and pathways that would be rare in hyperplastic lesions [13]. KA and SCC have drastically different molecular pathways and gene expression and can be distinguished from one another by these differences. One study utilized DNA microarrays to compare SCC with KA, demonstrating 1449 differentially expressed genes in KA in comparison with SCC (>5-fold change), with 908 genes upregulated and 541 genes downregulated [13]. When comparing KA with normal skin, the study found 2435 differentially expressed genes in KA in comparison with normal skin (>5-fold change), with 1085 genes upregulated and 1350 genes downregulated [13]. While the genes are too numerous to list here, several genes have changes in expression by factors ranging from dozens to hundreds [13]. These differences may be helpful if genetic analysis is available. 

SCC has been found to have relatively consistent BCL-2 levels and decreased BAK levels [45]. Regressing KAs have the opposite, with low levels of BCL-2 expression and increased BAK levels [45]. These ratios of pro- and anti-apoptotic proteins are reflective of the biological behavior of these two pathologies, with SCCs continuing to grow and KAs regressing.

Moreover, an overlap including amplifications of chromosomes such as 1p, 1q, 8q, 19, and deletion of 4q was noted. Some of these may have implications in tumorigenesis. *CMYC*, an oncogene, is located on 8q, and amplification of this gene may cause growth [46]. Another study found that cyclinD1 expression is present in both KAs and SCCs with staining found in both the nucleus and cytoplasm [47]. CyclinD1 regulates the cell cycle through the transition between G1 to S phase. Normally, CyclinD1 is transiently present in cells, but overexpression may result in excessive proliferation of skin cells. Likewise, it has been thought that a more gradual increase in CyclinD1 expression could contribute to the development of a tumor with a more aggressive nature [47]. Some studies have found a difference in the expression of BCL-XL and BAK between KAs and SCCs [37]. BCL-XL is an anti-apoptotic protein, while BAK is a pro-apoptotic protein. BCL-XL was found to be more positively stained in SCCs compared to KAs. BAK was found to be more strongly stained in KAs compared to SCCs. This has led to the speculation that apoptotic pathways may be activated in KAs resulting in the lesion’s regressive nature [37]. However, there have been other studies which reported no difference in expression of BCL-XL [48]. It has been further shown that there was no significant difference between KA and SCC for several markers such as tumor suppressors like TRAP-1, pRb, Cyld, p16, and p21 [37]. Yet there have been some conflicting results between studies as one study found no difference between p21 in these lesions [48]. HPV also seems to have a possible association with SCC and KAs. Previous studies reported HPV association in SCC, especially in sun-exposed areas [49,50]. It is possible that UV light may increase HPV activity and together, this can lead to the accumulation of mutations such as those in the tumor suppressor gene, p53 [49]. Yet another analysis reported no difference in HPV seen between SCCs and KAs [37].

Another study found that those who received BRAF inhibitors, such as vemurafenib, had common adverse events including KAs and cutaneous SCCs [51]. Thus, it is possible there may be involvement of the BRAF pathway for both these lesions. Molecularly, events which may result in inflammation of the skin such as external trauma or stress and pharmaceutical interventions may activate the MAP kinase pathway [52]. This pathway has roles in signaling and regulation of processes such as cell proliferation and differentiation [53]. The activation of this pathway could cause the development of both KAs and SCCs from abnormal cell growth. It seems that MAP kinase activity is increased with BRAF inhibition, which may be the cause of the increased incidence of KAs and SCCs in these patients. There may also be mutations which activate RAS through ultraviolet radiation and when exposed to BRAF inhibition, MAP kinase activity grows [52]. This could lead to a related pathogenesis between the two lesions.

### 3.4. Malignant Transformation of Keratoacanthoma to Squamous-Cell Carcinoma

The relationship between KA and SCC is somewhat controversial. Some studies presented cases of KA demonstrating metastatic behavior, which led to the term “keratoacanthomatous type of squamous-cell carcinoma”; however, this term is controversial [54].

### 3.5. Keratoacanthoma versus Squamous-Cell Carcinoma in CAP Protocols and WHO Books

In terms of staging systems for SCC, the most frequently used system in the United States is the Brigham and Women’s Hospital (BWH) and the eighth edition of the American Joint Committee on Cancer Recommendations (AJCC8) [55,56]. These systems are used to assess the pathologic features of the tumor necessary for appropriate management by clinicians.

Regarding grading systems, the World Health Organization (WHO) has made available documents for assessing lesions pathologically. The CAP no longer has a protocol for SCCs of the skin [57]. The most recent versions of the “WHO Classification of Tumors” online series by the WHO Evidence Synthesis and Classification branch describe important features of KA and SCCs in the fifth edition of the “Skin Tumors” publication. The text mentions that staging is not required for typical or regressed KAs, and any uncertainty in these lesions should be assessed by staging systems for SCCs [58]. The WHO Classification of Tumors takes the stance that these lesions are separate and should not be mixed in describing either one of them; for example, “squamous-cell carcinoma, keratoacanthoma-type” is listed as “not recommended” for reportable terminology of a KA, as this name describes a morphology of SCC [58].

## 4. Treatment of Keratoacanthoma versus Squamous-Cell Carcinoma

Generally speaking, the treatment for KAs and SCCs is the same: complete surgical excision, although it depends on lesion location, number of lesions, and suspicion of malignancy. Complete surgical excision gives certainty that the lesion is removed, and a higher-quality histologic examination can be performed, as a full-thickness sampling is preferred [1,3]. Realistically, a complete excision is not strictly necessary when treating a KA, as even a deep curettage can accomplish the task of treating a KA [59]. KAs may actually be triggered to transition to their natural regression phase by physical stimulation [4]. A biopsy itself may inadvertently accomplish this, and the lesion may regress rapidly after the biopsy is performed [4]. This feature of KAs does not change how they should be approached clinically, and it may be helpful information to provide to a patient so they may look out for a further reduction in tumor size post-biopsy and provide this information to the clinician. 

However, anything other than a complete-margin biopsy reduces the likelihood of a KA being correctly identified by microscopy, providing further justification for a complete excision [1,3]. Negative margins are ideal when evaluating the excised lesion, but positive margins do not result in increased recurrence rates for KAs [1]. Due to the guarantee that margins are examined during the procedure, Mohs micrographic surgery is an advantageous treatment method. Mohs micrographic surgery allows a more targeted locus in giant KAs or KA centrifugum, which have a higher likelihood of causing local tissue destruction or disfigurement [59]. Local or intralesional therapies such as imiquimod can cause spontaneous regression of KAs [60,61]. Systemic therapies such as methotrexate, cyclophosphamide, and 5-fluorouracil may also be utilized; however, they are not used as frequently as mechanical procedures such as excision, cryotherapy, or ablation [59]. Most systemic therapies are alternative treatments when there are multiple KAs with failed previous treatments or in cases of multiple eruptive KAs [59]. 

Regarding the treatment of SCC, the gold standard of treatment is surgical excision [55]. Most treatments utilized in SCC can be used to treat KAs. These conventional treatments include excision, Mohs micrographic surgery, radiation therapy, photodynamic therapy, immunotherapeutic, and chemotherapeutic agents [55]. Since SCC may metastasize to lymph nodes, clinical assessments and treatments must take this into account [62]. This is not a consideration for KA, and thus, the level of spread is far more concerning for SCC [58,59,63]. Highly mutated tumors become adequate candidates for new-generation immunotherapies as the likelihood that the tumor generates neoantigens increases [55]. Several differing treatments are used only in SCC (compared to KAs). One such therapy includes monoclonal antibodies serving as epidermal growth factor receptor (EGFR) inhibitors, such as panitumumab and cetuximab [62,64]. Another treatment for more advanced forms of SCC is checkpoint immunotherapy involving programmed cell death 1 inhibitors (PD-1) and programmed death-ligand 1 (PD-L1) inhibitors [55,64]. This recent advancement in PD-1 immune checkpoint inhibitors, such as cemiplimab and pembrolizumab, is a particularly promising and auspicious treatment, having been found to be superior to chemotherapy and EGFR inhibitors [65]. Since KAs and SCCs are different biological entities with different genetic pathways, these targeted immune therapies are simply not as effective in KAs.

## 5. Conclusions

In conclusion, significant progress has occurred since the era when KAs were poorly understood entities, in which even their cellular origin was a mystery. At this point in time, KAs are becoming well-understood. KAs should be regarded as a distinct entity, with histological and clinical characteristics being at the forefront of identification. Advances in genetic and molecular profiling have given answers to questions that have been posed for decades, and diagnostic accuracy will continue to improve as technology improves. Diagnostic hurdles such as ambiguous and varied architecture will become increasingly minor concerns as work on elucidating which IHC stains are most effective at separating KA and SCC continues. Collaboration and new findings will expedite this process, with the ultimate goal of an easily reproducible method in which staining can specify whether a sample is a KA or SCC.

Additionally, with the recent expansion of genomic testing, in the near future, we may expect a standard in which a sample that cannot be clearly diagnosed as KA and SCC by the traditional methods will be subjected to testing for genetic, molecular, and immune markers. While traditional gold-standard treatments such as mechanical excision continue to be the most utilized modes of treatment for both lesions, as research continues, more targeted treatment modalities will arise. These targeted treatments may cause greater patient and physician satisfaction, as their less invasive nature will lessen surgery’s legitimate health and aesthetic drawbacks. Continual investigation into optimal diagnostic and treatment guidelines and algorithms will lead to a greater number of favorable outcomes, serving our patients well.

At the rate at which new findings are being reported, the classic questions that have gone unanswered for these long periods of time will be concerns of the past, rendering themselves to be historical artifacts of an era in which we simply understood less about this fascinating pathology.

## Figures and Tables

**Table 1:** Differential diagnosis of keratoacanthoma based on clinical presentation.

Neoplastic Conditions	Infectious Diseases	Inflammatory Diseases
SCC (infundibular) Bowen disease Verrucous carcinoma Seborrheic keratosis Actinic keratosis KA-like SCC “KA with malignant transformation” Large-cell lymphomas Amelanotic melanoma	Sporotrichosis Cryptococcosis Blastomycosis Molluscum contagiosum	Hypertrophic form of discoid lupus erythematosus Lichen planus Halogenoderma Prurigo nodularis

Abbreviations: KA: keratoacanthoma; SCC: squamous-cell carcinoma.

**Table 2:** Table comparing the immunohistologic and immunocytologic markers between squamous-cell carcinoma and keratoacanthoma.

IHC Marker	Squamous-Cell Carcinoma	Keratoacanthoma
Ki67	High proliferation index (diffuse staining)	Low proliferation index (localized to the base)
CK17 [33]	Positive	Positive (central staining pattern)
E-cadherin [34]	Loss or reduced expression	Positive (retained)
BCL2 [35]	Typically, absent or weak	Positive
CD10 [36]	Negative	May be positive (localized to the base)
P16	Frequently overexpressed (aberrant pattern) in HPV-related SCC	Patchy (mosaic pattern)

Abbreviations: SCC: squamous-cell carcinoma.
